# P-508. Opportunities for Prep Prescription At Two Urban Academic Healthcare Institutions

**DOI:** 10.1093/ofid/ofae631.707

**Published:** 2025-01-29

**Authors:** Matthew Caputo, Jessica Ridgway, Joseph A Mason, Chad Achenbach, Moira McNulty

**Affiliations:** Northwestern University, Chicago, Illinois; University of Chicago Medicine, Chicago, Illinois; University of Chicago, Chicago, Illinois; Northwestern Memorial Hospital, Chicago, Illinois; University of Chicago, Chicago, Illinois

## Abstract

**Background:**

Pre-exposure prophylaxis (PrEP) use for HIV prevention remains unequal across groups vulnerable to HIV. Reasons for inequalities include both individual and healthcare level factors. We sought to understand opportunities for prescribing PrEP at two urban academic healthcare institutions in Chicago, Illinois.

**Methods:**

We analyzed electronic medical record data from both institutions including persons >18 years of age with >1 negative HIV test between 1/1/2015-12/31/2021 who had indications for PrEP. Data included demographics, social history, medications, sexually transmitted infections (STI), and encounters. Indications for PrEP included STI with gonorrhea, chlamydia, and/or active syphilis infection or injection drug use (IDU). Eligible encounters were those within six months after an STI, or as long as IDU was documented. We categorized encounters as inpatient, emergency department (ED), primary care, infectious disease (ID), obstetrics and gynecology/women’s health (OBGYN) and other outpatient settings.

**Results:**

In total, 9644 persons contributed 53031 encounters that resulted in 941 people with PrEP prescriptions. The two healthcare institutions had differing patient demographics, institution A had more 18-24 year olds (58.3% vs 31.3%), more African Americans (83.8% vs 27.9%), and more women (65.7% vs 46.3%). Institution B had more White (40.6% vs 7.1%) and Hispanic persons (14.0% vs 4.2%), and more men who have sex with men (MSM) (15.2% vs 3.3%). Institution A had more eligible encounters in the ED (30.8% vs 7.3%) as well as in infectious disease, inpatient, OBYGN, and primary care settings. Institution B had more eligible encounters in other outpatient settings (67.7% vs 18.7%). Institution B accounted for the majority of PrEP prescriptions (89.1%).

**Conclusion:**

Institution A contained persons historically underrepresented in PrEP prescriptions, while institution B had more encounters in other outpatient settings where ]PrEP prescriptions commonly occur. These factors are likely to be responsible for our findings. Future work will examine associations between these factors and the likelihood of PrEP prescriptions in a combined as well as institution specific models.

**Disclosures:**

**Jessica Ridgway, MD**, Gilead Sciences: Expert Testimony **Moira McNulty, MD**, Gilead Sciences, Inc.: Advisor/Consultant

